# The Roles of Variants in Human Multidrug Resistance (MDR1) Gene and Their Haplotypes on Antiepileptic Drugs Response: A Meta-Analysis of 57 Studies

**DOI:** 10.1371/journal.pone.0122043

**Published:** 2015-03-27

**Authors:** Hui Li, Bing Wang, Cheng Chang, Minghua Wu, Yun Xu, Yajun Jiang

**Affiliations:** 1 Department of neurology, Jiangsu Province Hospital of Traditional Chinese Medicine (TCM), Affiliated Hospital of Nanjing University of Chinese Medicine, Jiangsu, China; 2 Department of neurology, Huai’an Hospital of Traditional Chinese Medicine, Jiangsu, China; 3 Department of neurology, Nanjing Drum Tower Hospital affiliated with Medical School of Nanjing University, Nanjing, Jiangsu, China; Morehouse School of Medicine, UNITED STATES

## Abstract

**Objective:**

Previous studies reported the associations between the ATP-binding cassette sub-family B member 1 (ABCB1, also known as MDR1) polymorphisms and their haplotypes with risk of response to antiepileptic drugs in epilepsy, however, the results were inconclusive.

**Methods:**

The Pubmed, Embase, Web of Science, CNKI and Chinese Biomedicine databases were searched up to July 15, 2014. Pooled odds ratios (ORs) and 95% confidence intervals (CIs) were calculated using a fixed-effects or random-effects model based on heterogeneity tests. Meta-regression and Galbraith plot analysis were carried out to explore the possible heterogeneity.

**Results:**

A total of 57 studies involving 12407 patients (6083 drug-resistant and 6324 drug-responsive patients with epilepsy) were included in the pooled-analysis. For all three polymorphisms (C3435T, G2677T/A, and C1236T), we observed a wide spectrum of minor allele frequencies across different ethnicities. A significantly decreased risk of AEDs resistance was observed in Caucasian patients with T allele of C3435T variant, which was still significant after adjusted by multiple testing corrections (T vs C: OR=0.83, 95%CI=0.71-0.96, p=0.01). However, no significant association was observed between the other two variants and AEDs resistance. Of their haplotypes in ABCB1 gene (all studies were in Indians and Asians), no significant association was observed with AEDs resistance. Moreover, sensitivity and Cumulative analysis showed that the results of this meta-analysis were stable.

**Conclusion:**

In summary, this meta-analysis demonstrated that effect of C3435T variant on risk of AEDs resistance was ethnicity-dependent, which was significant in Caucasians. Additionally, further studies in different ethnic groups are warranted to clarify possible roles of haplotypes in ABCB1 gene in AEDs resistance, especially in Caucasians.

## Introduction

Epilepsy, one of the most common, chronic and disabling neurologic disorder, affects approximately 1% of the population worldwide, especially in developing countries.[1, 2] Although the prognosis for the most patients with epilepsy is good, 20%-30% of patients do not achieve seizure freedom despite multiple antiepileptic drugs (AEDs) treatment.[3–5] Recently, several factors have been identified to partly account for resistance to antiepileptic drugs, such as early onset, alcohol abuse, type of seizure, suboptimal dosing, poor drug compliance, and a high frequency of seizures in the diagnostic assessment period.[6–8] However, the exact mechanism of resistance remains poorly understood.

The ATP-binding cassette sub-family B member 1 (ABCB1, also known as MDR1) gene, which encodes human P-glycoprotein, can transport several AEDs.[9] In addition, previous studies have demonstrated that ABCB1 was also overexpressed in brain tissue from patients with refractory epilepsy, suggesting ABCB1 gene might be an important candidate gene responsible for refractory epilepsy.[10, 11] Siddiqui et al first reported that patients with drug-resistant epilepsy were more likely to have the CC genotype in C3435T variant, a well-known polymorphism in ABCB1 gene [12]. To date, an accumulating number of studies focused on the association between three polymorphisms (C3435T, G2677T/A, and C1236T) in ABCB1 gene and responsiveness to AEDs, however, the results were contradictory, mainly due to studies with ethnic differences, limited sample sizes, and inadequate statistical power.

To date, six meta-analyses focused on the association of ABCB1 variants with AEDs resistance. [13–18] However, the recent one included studies published up to 2012 (although the last search was updated in February 2013) and only investigated the association between one polymorphism (C3435T) and AEDs response.[15] The other recent meta-analysis reported the association in Chinese population.[18] Moreover, associations of the other variants in MRD1 gene (G2677T/A and C1236T variants) and the haplotypes with AEDs resistance were only analyzed in one research[17]. Since then, numerous additional studies reporting contradictory results were published.[19–26] Hence, we conducted a meta-analysis to clarify the associations of three polymorphisms in ABCB1 gene and their haplotypes with responsiveness to AEDs in patients with epilepsy.

## Materials and Methods

### Search strategy

A comprehensive electronic search involving Pubmed, Embase, and Web of science, CNKI (China National Knowledge Infrastructure) and Chinese Biomedicine Databases was carried out to identify the association of ABCB1 gene polymorphisms with antiepileptic drug response in patients with epilepsy, using the following search terms: ‘‘multidrug resistance 1 gene” or “ABCB1” or “MDR1” or “C1236T” or “C3435T” or “G2677T/A” or “rs1045642” or “rs1128503” or “rs2032582”, “polymorphism” or “variant” or “SNP”, AND “epilepsy” or “seizure” (the last search update was 15 July 2014). In addition, the bibliographies of all retrieved articles were hand-searched for additional potential studies.

### Inclusion and exclusion criteria

The studies were eligible for the meta-analysis if they meet the following criteria: 1) case-control or cohort design 2) reported the association between MRD1 polymorphisms and drug response in epilepsy patients 3) phenotypes of drug response were clearly defined. Studies were excluded for the following exclusion criteria: 1) compared drug-resistant patients with healthy individuals 2) did not describe the definition of drug response 3) comments, review articles, or articles only with an abstract.

### Data extraction

Two independent investigators extracted the following data from each included study: first author, publication year, ethnicity (Caucasians, Asians, or Africans), age and sample size of patients with drug-resistance and drug responsiveness, definition of drug-resistance and drug responsiveness, allele and genotype distribution in drug-resistant and drug-responsive patients. Disagreements were resolved by consulting with a third author. In addition, articles that reported results from more than one subpopulation or adults and children separately were considered as separate studies.

### Statistical analysis

The overall association between three polymorphisms in ABCB1 gene (C3435T, C1236T, and G2677T/A) and antiepileptic drug-resistance was assessed by odds ratios (ORs) with 95% confidence intervals (CIs). The significance of the pooled OR was determined by the Z-test, and the P values were adjusted using Bonferroni correction by the number of compared SNPs. (P = 0.05/3 = 0.017). For simplification of the analysis of the G2677T/A variant, the A allele was included with the T allele as previously described.[17] Chisquare based Q test and I^2^ test were carried out to assess the heterogeneity between studies, which was considered significant when P<0.10.[27, 28] A random effects model (DerSimonian-Laird) was used when the significant heterogeneity existed, otherwise, a fixed model was used (Mantel-Haenszel). [29, 30] Moreover, subgroup analysis and meta-regression analysis were carried out to explore the possible heterogeneity among different kinds of studies.[31] Finally, the Galbraith plot was used to spot the outliers as the possible major sources of heterogeneity. [32, 33]

To assess the stability of the results, sensitivity analysis by sequential removal of each study was carried out. Moreover, sensitive analysis limited to English language studies or studies in HWE was also performed to assess the stable of results. Cumulative meta-analyses for each polymorphism were also performed to investigate the trend and the stability of risk effect as evidence accumulated over time, through assortment of studies with publication time.

Begg’s Funnel plots and Egger regression asymmetry test were performed to assess the potential publication bias, and a p-value from the Egger’s test less than 0.05 was considered statistically significant.[34, 35] If there was some evidence of significant publication bias, ORs and 95% CIs would be adjusted by Duval and Tweedie’s nonparametric trim and fill methods.[36] All statistical analyses were performed by STATA software, version 12 (StataCorp LP, College Station, Texas).

## Results

### Study characteristics

**Fig 1:**
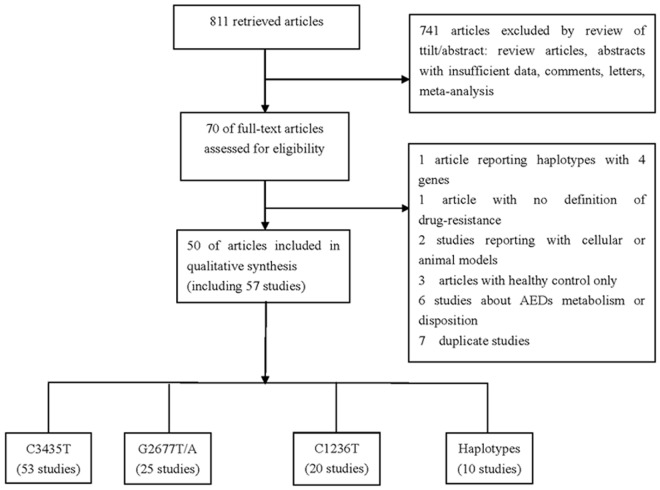
Study selection procedures for a meta-analysis of MDR1 gene polymorphisms (C3435T, G2677T, and C1236T) with AEDs resistance. AEDs: antiepileptic drugs; ABCB1: ATP-binding cassette sub-family B member 1.

The literature review identified 811studies, of which 741 articles were excluded by review of titles and abstracts and 20 studies were excluded after assessing of full-text articles (S1 Table). Finally, a total of 50 articles were eligible for the pooled analysis (Table 1).[12, 19–26, 37–83] Among these, 2 articles reported 6 subpopulations and 3 articles investigated adults and children, respectively. Finally, a total of 57 studies involving 6083 drug-resistant and 6324 drug-responsive patients with epilepsy were eligible for the meta-analysis. Among all eligible studies, 53, 25 and 20 studies reported the data on C3435T, G2677T, and C1236T variant, respectively. In addition, 10 studies investigated the association of ABCB1 haplotype (C1236T–G2677T–C3435T loci) with AEDs response. The details for the literature search were shown in Fig. 1.

**Table 1:** Characteristics of studies analyzed for meta-analysis of association between the C1236T, G2677T, and C3435T variants in ABCB1 gene and drug response in patients with epilepsy.

Author and year	Ethnicity	Sample size		Phenotypic definition	
		DNR	DR	DNR	DR
Siddiqui, 2003	Caucasians	200	115	≥4 seizures (1 year), >3 AEDs	No seizure (1 year)
Soranzo, 2004	Caucasians	286	135	≥4 seizures (1 year), >3 AEDs	No seizure (1 year)
Tan, 2004	Caucasians	401	208	≥4 seizures (1 year), >3 AEDs	No seizure (1 year)
Sills, 2005	Caucasians	230	170	≥1 seizure (1 year), ≥2 AEDs	No seizure (1 year)
Kim, 2006	Asians	99	108	≥4 seizures (1 year), ≥3 AEDs	No seizure (1 year)
Ozgon, 2006	Caucasians	44	53	≥4 seizures (6 months), CBZ	No seizure (1 year)
Seo, 2006	Asians	126	84	≥1 seizure (1 year), CBZ	No seizure (1 year)
Kim, 2006	Asians	63	108	≥4 seizures (1 year), >3 AEDs	No seizure (1 year)
Chen, 2007	Asians	50	164	≥1 seizure/month (1 year), ≥2 AEDs	No seizure (1 year)
Lu, 2007	Asians	72	62	<50% reduction in seizure frequency (1 year)	≥50% reduction in seizure frequency (1 year)
Leschziner, 2007	Caucasians	73	76	≥4 seizures (1 year), >2 AEDs	not fulfilling DNR criteria
Ebid, 2007	Caucasians	60	37	≥1 seizure (3 months), PHT	No seizure (3 months)
Hung, 2007	Asians	114	213	>10 seizures (1 year), ≥2 AEDs	No seizure (2 years)
Kwan, 2007	Asians	221	297	≥1 seizure/month (1 year), ≥2 AEDs	No seizure (1 year)
Shahwan, 2007	Caucasians	198	242	<50% reduction in seizure frequency (1 year), ≥3 AEDs	≥50% reduction in seizure frequency (1 year)
Wang, 2008	Asians	40	40	≥4 seizures/month (2 years)	No seizure (1 year), 1 AED
Gao, 2009	Asians	70	62	>1 seizure/month (6 months), >2 AEDs	No seizure (1 year)
Kim, 2009	Asians	198	193	≥4 seizures (1 year), ≥3 AEDs	No seizure (1 year)
Kwan, 2009	Asians	194	270	≥1 seizure/month (1 year), ≥2 AEDs	No seizure (1 year)
Lakhan, 2009	Indian	94	231	≥4 seizures (1 year), 3 AEDs	No seizure (1 year)
Szoeke,2009	Caucasians	133	152	≥1 seizure (1 year)	No seizure (1 year)
Szoeke,2009	Caucasians	64	148	≥1 seizure (1 year)	No seizure (1 year)
Szoeke,2009	Asians	11	34	≥1 seizure (1 year)	No seizure (1 year)
Ufer, 2009	Caucasians	118	103	Receving second-line drug due to non-response or adverse reactions to initial AED treatment	Responders to the first-line drug
Vahab, 2009	Asians	113	129	<6 months terminal remission, ≥2 AEDs	No seizure (1 year)
Von Stülpnagel, 2009	Caucasians	160	71	failing to be seizure-free, and/or having epilepsy surgery, ≥3AEDs	seizure-free ≤6 months), ≤ 2 AEDs
Zheng, 2009	Asians	31	33	≥4 seizures/month, 2 years	No seizure (1 year)
Grover, 2010	Indian	95	133	≥1 seizure (10 months)	No seizure (10 months)
Jin, 2010(C1236T)	Asians	108	122	<50% reduction in seizure frequency (12 months), ≥2 AEDs	≥50% reduction in seizure frequency (1 year)
Jin, 2010(C3435T)	Asians	108	122	<50% reduction in seizure frequency (12 months), ≥2 AEDs	≥50% reduction in seizure frequency (1 year)
Maleki, 2010	Caucasians	132	200	≥1 seizure/month or ≥10 seizures (12 months), ≥2 AEDs	No seizure (1 year), 1 AED
Maleki, 2010	Caucasians	132	200	≥1 seizure/month or ≥10 seizures (12 months), ≥2 AEDs	No seizure (1 year), 1 AED
Sánchez, 2010	Caucasians	52	28	≥4 seizures (1 year), >3 AEDs	No seizure (1 year)
Sánchez, 2010	Caucasians	126	83	≥4 seizures (1 year), >3 AEDs	No seizure (1 year)
Dong, 2010	Asians	157	193	≥4 seizures (1 year), ≥2 AEDs	No seizure (1 year)
Di, 2011	Asians	91	79	≥1 seizure/month (2 years), ≥2 AEDs	<1 seuzure/month (2 years)
Dong, 2011	Asians	95	80	<50% reduction in seizure frequency (1 year)	≥50% reduction in seizure frequency (1 year)
Haerian, 2011	Asians	131	146	≥1 seizure/month (1 year), CBZ or VPA	No seizure (1 year), CBZ or VPA
Haerian, 2011	Asians	67	93	≥1 seizure/month (1 year), CBZ or VPA	No seizure (1 year), CBZ or VPA
Haerian, 2011	Asians	125	123	≥1 seizure/month (1 year), CBZ or VPA	No seizure (1 year), CBZ or VPA
Meng, 2011	Asians	24	60	<50% reduction in seizure frequency (1 year), CBZ	≥50% reduction in seizure frequency (1 year), CBZ
Sayyah, 2011	Caucasians	4	10	≥1 seizure/month or ≥10 seizures (1 year), ≥2 AEDs	No seizure (1 year), 1 AED
Sayyah, 2011	Caucasians	128	190	≥1 seizure/month or ≥10 seizures (1 year), ≥2 AEDs	No seizure (1 year), 1 AED
Sporiš, 2011	Caucasians	57	48	≥1 seizure/month (1 year), ≥2 AEDs	No seizure (1 year)
Wang, 2011	Asians	85	71	≥4 seizures/month, 2 years	No seizure (1 year)
Qu, 2012	Asians	217	320	≥4 seizures (1 year), ≥3 AEDs	No seizure (1 year)
Sterjev, 2012	Caucasians	68	94	≥4 seizures (1 year), CBZ	No seizure (1 year), CBZ
Yang, 2012	Asians	23	26	<50% reduction in seizure frequency (1 year)	≥50% reduction in seizure frequency (1 year),
Buathet, 2013	Asians	68	36	epileptic seizures continued	No seizure (1 year), or 3 times longer than the previous interparoxysmal period
Emich-Widera, 2013	Caucasians	60	25	epileptic seizures continued, in monotherapy or polytherapy	No seizure (1 year), or 3 times longer than the previous interparoxysmal period
Huang, 2013	Asians	30	38	≥1 seizure (1 year)	No seizure (1 year), or 3 times longer than the previous interparoxysmal period
Subenthiran, 2013	Asians	162	152	seizures continued, in polytherapy	No seizure (1 year), CBZ
Balan, 2014	Indian	259	201	≥12 seizures (1 year), ≥2 AEDs	No seizure (≥1 year)
Saygi,2014	Caucasians	59	60	≥4 seizures (1 year), ≥3 AEDs	No seizure (1 year)
Seven, 2014	Caucasians	69	83	≥4 seizures (1 year), 3 AEDs	No seizure (1 year)
Shaheen, 2014	Indian	128	92	a poor clinical outcome and recurrent seizure events in epileptic patients	No seizure (1 year)

CBZ: carbamazepine; PHT: phenytoin; VPA: sodium valproate; AEDs: antiepileptic drugs; n.r.: not reported. DNR: Drug resistance; DR: Drug responsiveness.

In the drug-resistant patients, we observed that T allele frequency of C3435T variant was significant higher in Caucasians (49.75%±8.24%) than those in Asians (40.47%±8.09%), which was also observed in drug-response and overall patients with epilepsy. (S2 Table) For G2677T polymorphism, a similar distribution of the T/A allele was observed across Caucasians and Asians both in drug-resistant and drug-responsive patients with epilepsy. For C1236T variant, T allelic frequency was similar in Indians (58.95%±4.85%) and Asians (63.46%±4.96%) but lowest in Caucasians (44.85%±7.88%, p = 0.01).

### Association of C3435T variant with antiepileptic drug-resistance

**Fig 2:**
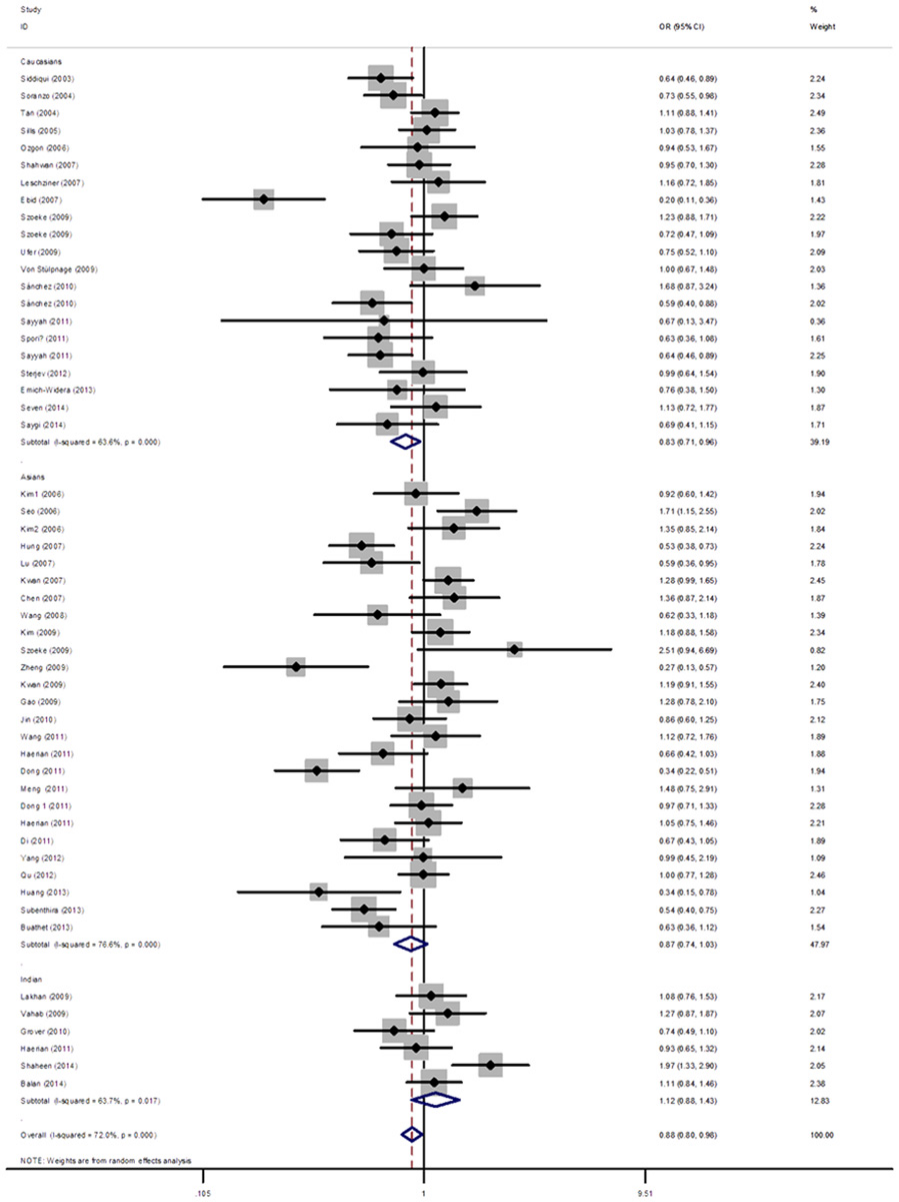
Odds ratio (OR) estimates for the association between the ABCB1 C3435T polymorphism and AEDs resistance. The sizes of the squares reflect theweighting of the included studies. Bars represent 95% CIs. The center of the diamond represents the summary effect; left and right points of the diamond represent the 95% CI. AEDs: antiepileptic drugs; CI: confidence interval; ABCB1: ATP-binding cassette sub-family B member 1.

The fifty six studies that investigated the correlation between C3435T variant and drug response included 12407 patients with epilepsy (6083 drug-resistance and 6324 drug-response). Although a significant association of C3435T polymorphism with antiepileptic drug-resistance was observed in the allelic and genetic models (T vs C: OR = 0.88(0.79, 0.98), p = 0.02; TT vs CC: T OR = 0.79(0.63, 0.98), p = 0.03) (Table 2 and Fig. 2), the significance was removed after adjusted by multiple testing corrections. In the subgroup analysis stratified by ethnicity, we observed a significantly decreased risk of drug-resistance in Caucasians (OR = 0.83(0.71, 0,96), p = 0.01), but not among Asians (OR = 0.87(0.74, 1.03), p = 0.11). However, when stratifying by age, no statistical association between C3435T variant and drug-responsiveness was observed in children or adult patients with epilepsy. (Table 2)

**Table 2:** Summary odds ratios and heterogeneity of the C3435T polymorphism in ABCB1 gene on drug response in patients with epilepsy stratified by age, language, ethnicity, sample size and date of publication.

	No	T vs C			TT vs CC			TC vs CC			TT+TC vs CC			TT vs TC+CC		
		OR(95%CI)	P	Ph	OR(95%CI)	P	Ph	OR(95%CI)	P	Ph	OR(95%CI)	P	Ph	OR(95%CI)	P	Ph
**Total**	53	**0.88(0.79, 0.98)**	0.02	<0.01	**0.79(0.63,0.98)**	0.03	<0.01	0.89(0.77,1.02)	0.1	<0.01	0.84(0.72,0.98)	0.03	<0.01	0.87(0.75,1.01)	0.08	<0.01
**English-language publications**	42	0.93(0.83,1.03)	0.18	<0.01	0.87(0.69,1.09)	0.22	<0.01	0.94(0.82,1.09)	0.45	<0.01	0.91(0.77,1.07)	0.25	<0.01	0.91(0.78,1.07)	0.25	<0.01
**All in HWE**	40	0.87(0.77,0.99)	0.03	<0.01	0.77(0.60,0.99)	0.04	<0.01	0.91(0.78,1.05)	0.19	<0.01	0.86(0.72,1.02)	0.08	<0.01	0.84(0.72,0.99)	0.03	<0.01
**Ethnicity**																
Caucasians	21	**0.83(0.71,0.96)**	0.01	<0.01	**0.67(0.50,0.91)**	0.01	<0.01	**0.78(0.63,0.96)**	0.02	0.06	**0.74(0.58,0.93)**	0.01	<0.01	**0.83(0.69,0.99)**	0.04	0.05
Asians	26	0.87(0.74,1.03)	0.11	<0.01	0.78(0.55,1.11)	0.17	<0.01	0.90(0.74,1.10)	0.31	<0.01	0.84(0.68,1.05)	0.13	<0.01	0.85(0.64,1.12)	0.25	<0.01
Indian	6	1.12(0.88,1.43)	0.34	0.02	1.38(0.76,2.49)	0.29	0.024	1.31(0.91,1.88)	0.14	0.31	1.33(0.85,2.09)	0.21	0.09	1.12(0.80,1.57)	0.51	0.03
**Age**																
Children	11	1.05(0.89,1.23)	0.57	0.32	1.03(0.72,1.48)	0.85	0.29	1.06(0.83,1.37)	0.63	0.73	1.06(0.83,1.34)	0.66	0.50	1.09(0.86,1.38)	0.48	0.53
Adults	12	0.78(0.58,1.03)	0.08	<0.01	0.59(0.33,1.07)	0.08	<0.01	0.80(0.58,1.11)	0.18	<0.01	0.71(0.49,1.04)	0.08	<0.01	0.70(0.45,1.09)	0.11	<0.01
**Sample size**																
>200	28	0.95(0.85,1.07)	0.43	<0.01	0.94(0.73,1.20)	0.62	<0.01	0.93(0.81,1.07)	0.33	0.03	0.92(0.78,1.09)	0.33	<0.01	0.98(0.83,1.16)	0.79	<0.01
≤200	25	**0.78(0.63,0.96)**	0.02	<0.01	0.57(0.38,0.88)	0.01	<0.01	0.79(0.58,1.07)	0.13	<0.01	0.71(0.52,0.97)	0.03	<0.01	0.69(0.51,0.92)	0.01	<0.01
**Publication years**																
>2010	30	**0.83(0.70,0.98)**	0.03	<0.01	0.69(0.49,0.97)	0.03	<0.01	0.90(0.74,1.11)	0.34	0.04	0.82(0.65,1.04)	0.10	<0.01	0.76(0.60,0.98)	0.03	<0.01
≤2010	23	0.92(0.80,1.05)	0.23	<0.01	0.86(0.64,1.15)	0.30	<0.01	0.88(0.73,1.06)	0.19	<0.01	0.85(0.69,1.05)	0.14	<0.01	0.95(0.79,1.15)	0.62	<0.01

CI: confidence interval; HWE: Hardy-Weinberg equilibrium; No: Number of studies; OR: odds ratio; P_h_: P-value for heterogeneity tests.

### Association of G2677T variant with antiepileptic drug-resistance

In the pooled analysis of 25 studies for G2667T polymorphism, no significant association was recorded (T vs G: OR = 0.95(0.80, 1.12), p = 0.52). (S3 Table) Similarly, sub-analysis showed no significant association between G2677T polymorphism and responses to AEDs in Asian, Caucasian or Indian subgroup, child or adult subgroup, large or small sample size, and publication after 2010 or before 2010. (OR = 0.90(0.65, 1.27), p = 0.56; OR = 0.92(0.76, 1.11), p = 0.39; OR = 1.05(0.89, 1.23), p = 0.57; OR = 1.14(0.95, 1.36), p = 0.16; OR = 0.78(0.53, 1.15), p = 0.21; OR = 0.90(0.75, 1.09), p = 0.28; OR = 1.14(0.83, 1.55), p = 0.42; OR = 0.82(0.59, 1.14), p = 0.25; OR = 1.06(0.93, 1.20), p = 0.37, respectively)

### Association of C1236T variant with antiepileptic drug-resistance

The associations of ABCB1 C1236T variant with responses to AEDs were investigated in 20 studies, and the results presented in S4 Table. The results showed that no obvious association was observed between C1236T variant and antiepileptic drug-resistance in any genetic models in overall populations (T vs C: OR = 0.97(0.90, 1.06), p = 0.56; TT vs CC: OR = 0.96(0.81, 1.14), p = 0.67; TC vs CC: OR = 1.01(0.86, 1.20), p = 0.88; TT+TC vs CC: OR = 0.99(0.84, 1.16), p = 0.88; TT vs TC+CC: OR = 0.97(0.86, 1.09), p = 0.63) or in the subgroup analysis by ethnicity, age of patients, sample size, and publication years.

### Association of ABCB1 “C1236T/G2677T/C3435T” haplotypes with antiepileptic drug-resistance

We further investigated the association of the haplotypic combinations of C1236T, G2677T, and C3435T variants with AEDs response, which included 10 studies involving 1113 drug-resistant and 1454 drug-responsive patients. (S5 Table) Haplotype pooled analysis showed no significant association of the three haplotypic models (TTT vs CGC: OR = 1.04(0.82, 1.32), p = 0.72; TTT vs non-TTT: OR = 1.31(0.94, 1.81), p = 0.11; and non-CGC vs CGC: OR = 0.83(0.51, 1.35), p = 0.46) with the response to AEDs in overall populations. In addition, no statistical association was detected in two subgroups (Indians and Asians) when stratified by ethnicity. Heterogeneity was observed in major models in overall populations and subgroups stratified by ethnicity. (S6 Table)

### Tests for heterogeneity

There was significant heterogeneity in most comparisons of C3435T and G2677T variants in overall populations. (T vs C for C3435T: P_h_<0.01, and T vs G for G2677T: P_h_<0.01) The heterogeneity was removed in the subgroup of Caucasians, Indian, children, small sample size, publications before 2010 for C3435T variant, but only in children subgroup for G2677T variant. Then, meta-regression was performed to assess the source of heterogeneity for allelic model by ethnicity, age of patients, sample size and year of publications. However, the results showed that no source contributed to the substantial heterogeneity. In addition, Galbraith plot for observing heterogeneity identified 1 study in G2677T polymorphism and 10 studies in C3435T polymorphism as outliners, which were the potential origin of heterogeneity. (S1 Fig.) For the analysis of C1236T polymorphism, we did not observe any heterogeneity in all allelic and genotypic models (T vs C: P_h_ = 0.51, TT vs CC: P_h_ = 0.69, TC vs CC: P_h_ = 0.81, dominant model: P_h_ = 0.75, and recessive model: p = 0.65).

### Sensitivity and cumulative analysis

Sensitivity analysis was performed for three polymorphisms (C3435T, G2677T, and C1236T) by sequential removal of each study, the results of which showed that no single study qualitatively changed the pooled ORs, suggesting that the results of this meta-analysis are highly stable. (S2 Fig.) Moreover, sensitivity analyses limited to English language studies showed the ORs did not change after excluding Chinese-language studies. There were 12, 6 and 3 studies which deviated from HWE for C3435T, G2677T, and C1236T variant respectively, whereas the pooled ORs were not materially altered when these studies were excluded. (Table 2, S3 Table, and S4 Table) In the cumulative meta-analysis, the results showed that the pooled OR tended to be stable, whereas the association was still not significant with accumulation of more data over time. (S3 Fig.)

### Publication bias

**Fig 3:**
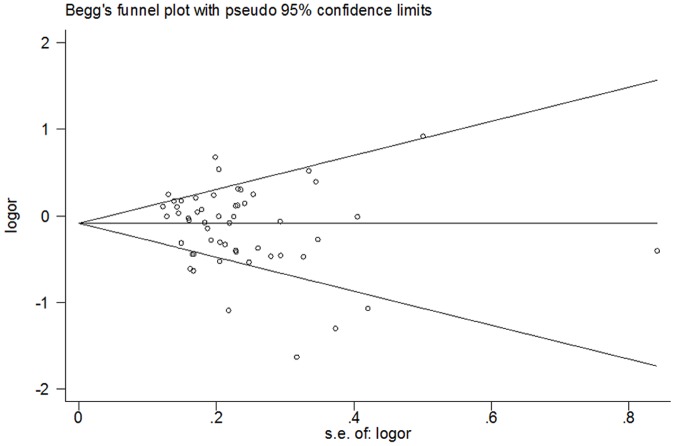
Begg's funnel plots for publication bias test of the association between the ABCB1 C3435T polymorphism and AEDs resistance. No significant funnel asymmetry that could indicate publication bias was observed. The horizontal line in the funnel plot indicates the random-effects summary estimate, and the sloping lines indicate the expected 95% confidence interval for a given standard error, assuming no heterogeneity between studies. Log(OR) is the natural logarithm of the odds ratio. AEDs: antiepileptic drugs; ABCB1: ATP-binding cassette sub-family B member 1.

Begg’s funnel plot and Egger’s test were performed to assess potential publication bias of literatures. The shapes of the Begg’s funnel plots did not reveal any signs of obvious asymmetry. (Fig. 3) In addition, Egger’s test did not show statistical significance for publication bias (P = 0.111 for T vs C in C3435T, p = 0.679 for T vs G in G2677T, and p = 0.218 for T vs C in C1236T).

## Discussion

ABCB1, a kind of multidrug transporters, belongs to the adenosine triphosphate (ATP)-binding cassette super family. Protein encoded by ABCB1 gene is a 170-kDa transmembrane glycoprotein expressed in blood–brain barrier [78], which acts as a drug-efflux pump, involved in absorption and excretion. ABCB1 may pump AEDs back from brain into the blood and reduce antiepileptic drug concentration around neurons in the seizure focus, contributing to AED resistance. Accumulated animal and clinical studies suggested that ABCB1 might be a possible factor responsible for refractory epilepsy [10–12,79]. Kimchi-Sarfaty C et al showed that polymorphisms in ABCB1 might affect the timing of cotranslational folding and insertion of P-gp into the membrane, thereby altering the structure of substrate and inhibitor interaction sites.[80] Recently, attention has been focused on the genetic mutations in ABCB1 gene that affect responsiveness to AEDs in patients with epilepsy, whereas previous studies reporting the association between ABCB1 gene polymorphisms and response to AEDs provided inconclusive results.

In this comprehensive meta-analysis involving 57 studies, we found that patients with a T allele in C3435T polymorphism had a significantly decreased risk of drug-resistance in Caucasians, but not among Asians and Indians. No statistical associations were observed of the other two variants in ABCB1 gene (G2677T and C1236T) with risk of drug-resistance in overall populations or subgroup analysis by ethnicity, sample size, date of publication, and age of patients. Additionally, the pooled analysis did not reveal evidence of the association between haplotypes of these three loci and AEDs responsive.

Genetic polymorphism often varies between ethnic groups, which was one of the factors that might affect the results. Distribution of allelic frequencies in ABCB1 C3435T variant also displays an ethnic difference. [12,37,47] In the present study, we showed that T alleles in C3435T variant were more common in Caucasians than those in Asians, but lower than those in Indians. Moreover, stratified meta-analysis showed an ethnic-dependent susceptibility to AEDs of C3435T polymorphism, which was significant associated with AEDs resistance in Caucasian population, but not in Asian and Indian subgroups. These observations might be attributed to that different populations are under distinct environmental or cultural pressures.

Age might be another factor that influences the AEDs response. [59,81] Types of AEDs are often be different regarding patient age, use of valproate and carbamazepine is more frequently in children, while adults are more usually treated with phenytoin or phenobarbital.[59] Moreover, children often require higher dosages than those recommended for adults attributed to the more rapid clearance and variability in elimination kinetics of AEDs.[81] In our meta-analysis, however, when stratified by age, no significant associations were observed in children or adults subgroups. Moreover, for C3435T variant, majority of studies included in our meta-analysis reported mixed age of patients, range from children to adults (30/53). On the other hand, only 12 and 11 studies investigated adult and child patients, respectively. Thus, the small sample size might contribute to, at least partially, the lack of association in the adult and child subpopulation.

Previous study demonstrated that C3435T in exon 26 is a silent variant (no amino acid change), it may influence the AEDs drug response by linkage disequilibrium with another variant, including G2677T and C1236T variants in ABCB1 gene.[82] Linkage disequilibrium was defined as the association between different variant alleles at multiple polymorphic sites in the genome. Here, pooled analysis of 2567 patients did not showed any associations of haplotypes (TTT vs CGC, TTT vs non-TTT, and non-CGC vs CGC) with drug-resistance in patients with epilepsy in overall populations or Asian and Indian subgroups. However, studies investigating the association between C1236T/G2677T/C3435T haplotypes and AEDs resistance in Caucasians were not reported, thus further studies are required to explore whether ABCB1 haplotypes had an ethnic-dependent effect similar to C3435T polymorphisms.

Heterogeneity was significant for the most comparisons of C3435T and G2677T variants in overall population. To identify the potential source of heterogeneity, we performed subgroup analysis, meta-regression and Galbraith plot analysis. The results showed that heterogeneity was removed or significantly decreased in Indian and children subgroup group for C3435T variant, and in Caucasian, Indian, and children for G2677T variants. These might be attributed to different genetic backgrounds, different environments, different lifestyles or different AEDs among different ethnicities and ages. However, meta-regression analysis did not found any potential source contributing to the heterogeneity.

In addition, variability in the definitions of drug-response and drug-resistance might also contribute to the significant heterogeneity. Of all included studies, the follow-up time ranged from 3 months to 2 years (most studies followed 1 year), whereas the new definition of treatment outcome from International League Against Epilepsy (ILAE) reported that the shortest follow-up period was 12 months.[83] Moreover, the new definition of drug resistant epilepsy was failure of adequate trials of two tolerated and appropriately chosen and used AED schedules to achieve sustained seizure freedom. In all eligible studies, types of AEDs were also variable (from only 1 to more than 3). Moreover, drug-response was defined as patients with no seizure for more than three times the pretreatment interseizure interval or 12 months, whichever is longer,[83] which was applied only in three studies.

Several limitations need to be considered for interpretation of our results. First, most AED responses are influenced by an interaction of multiple factors: environmental or patient-related factors and characteristics of the epilepsy itself, and genetic factors, statistical adjustment for individual level factors were not carried out for the insufficient data. Second, types of seizures might also cause variety in AEDs types, dosage, and drug response, a subgroup analysis by types of seizures was necessary in further meta-analysis.[1] Finally, the definition used to classify patients as being drug-resistance has varied in different studies, which may contribute to the variations in the results.

In conclusion, the present systematic review and meta-analysis involving 57 studies with 12407 PWE suggested that C3435T variant, but not G2667T or C1236T variant, might play a role in altered AEDs response ethnicity-dependently. In addition, the association between haplotypes of ABCB1 variants and AEDs response were reported only in Indians and Asians, thus, further studies in different ethnic groups are warranted to clarify possible roles of haplotypes in ABCB1 gene in AEDs resistance, especially in Caucasians. As the new definition of the ILAE has been published, variability in the definitions of AEDs resistance in further studies should be avoided. Finally, these findings might provide predictive genetic markers for antiepileptic drug effectiveness or resistance in individual patients or a possibility for future pharmacogenetically-adjusted dosing of AED, which could be translated into clinical practice.

## Supporting Information

S1 PRISMA Checklist PRISMA checklist. (DOC)

S1 Table Reasons for exclusion of full-texts. (DOC)

S2 Table Distribution of allelic frequencies (T allele in C1236T variant, T allele in G2677T variant, and T allele in C3435T variant) across different ethnic groups. (DOC)

S3 Table Summary odds ratios and heterogeneity of the G2677T polymorphism in ABCB1 gene on drug response in patients with epilepsy stratified by age, ethnicity, sample size and date of publication. (DOC)

S4 Table Summary odds ratios and heterogeneity of the C1236T polymorphism in ABCB1 gene on drug response in patients with epilepsy stratified by age, ethnicity, sample size and date of publication. (DOC)

S5 Table Distribution of haplotypic frequencies of the ACBC1 polymorphisms at C1236T, G2677T and C3435T. (DOC)

S6 Table Summary odds ratios and heterogeneity of haplotypic comparisons in ABCB1 gene on drug response in patients with epilepsy stratified by ethnicity. (DOC)

S1 Fig Galbraith plots of MDR1 C3435T polymorphism and AEDs resistance. The regression runs through the origin interval (central solid line). The 95% confidence interval is between the two outer parallel lines at two units above and below the regression line.
(TIF)

S2 Fig Sensitivity analysis on the associations of MDR1 C3435T polymorphism with AEDs resistance. Results were computed by omitting each study (left column) in turn, Bars: 95% confidence interval.
(TIF)

S3 Fig Cumulative meta-analysis of associations between MDR1 C3435T polymorphism and AEDs resistance. The circles and horizontal lines show the accumulation of estimates as results from each study at the end of each year were added.
(TIF)
