# Supplementary material for: The Roles of Variants in Human Multidrug Resistance (MDR1) Gene and Their Haplotypes on Antiepileptic Drugs Response: A Meta-Analysis of 57 Studies
Source: PLoS One. 2015 Mar 27;10(3):e0122043. doi: 10.1371/journal.pone.0122043 (PMC4376792; doi:10.1371/journal.pone.0122043)
Supplement: S1 Table — (DOC) [file pone.0122043.s002.doc]

**S1_Table. Reasons for exclusion of full-texts**

| **Author and year** | **Title** | **Reasons for exclusion** |
| --- | --- | --- |
| Zimprich, 2004[1] | Association of an ABCB1 gene haplotype with pharmacoresistance in temporal lobe epilepsy | Controls are healthy volunteers |
| Leschziner, 2006[2] | Exon sequencing and high resolution haplotype analysis of ABC transporter genes implicated in drug resistance | No sufficient data |
| Haerian, 2011[3] | Association between ABCB1 polymorphism and response to sodium valproate treatment in Malaysian epilepsy patients | Containing overlapping data |
| Dericioglu, 2008[4] | Multidrug resistance in patients undergoing resective epilepsy surgery is not associated with C3435T polymorphism in the ABCB1 (MDR1) gene | Controls are healthy volunteers |
| Mosyagin, 2008[5] | Association of ABCB1 genetic variants 3435C>T and 2677G>T to ABCB1 mRNA and protein expression in brain tissue from refractory epilepsy patients | Expression of mRNA in patient brain |
| Alpman, 2010[6] | Multidrug Resistance 1 (MDR1) Gene Polymorphisms in Childhood Drug-Resistant Epilepsy | Controls are healthy volunteers |
| Dong, 2010[7] | Correlation between C1236T polymorphism in ABCB1 gene and response of antiepileptic drug treatment in Chinese epileptic children | Containing overlapping data |
| Hung, 2008[8] | Functional evaluation of polymorphisms in the human ABCB1 gene and the impact on clinical responses of antiepileptic drugs | Cellular experiments |
| Hung, 2008[9] | Role of MDR1 C3435T and GABRG2 C588T Gene Polymorphisms in Seizure Occurrence and MDR1 Effect on Anti-Epileptic Drug (Phenytoin) Absorption | No identification of drug response |
| Guo, 2010[10] | Association study of C3435T genetic polymorphism ofMDR1 gene with efficacy of carbamazepine | Containing overlapping data |
| Tate, 2005[11] | Genetic predictors of the maximum doses patients receive during clinical use of the anti-epileptic drugs  carbamazepine and phenytoin | Investigation of dose of AEDs |
| Wang, 2008[12] | The relationship between the single nucleotide polymorphisms of multidrug resistance gene 1, brain derived neurotrophic factor gene and childhood drug resistance epilepsy | Containing overlapping data |
| Puranik, 2013[13] | Association of carbamazepine major metabolism and transport pathway gene polymorphisms and pharmacokinetics in patients with epilepsy | Investigation of AEDs metabolism |
| Grover, 2012[14] | Genetic association analysis of transporters identifies ABCC2 loci for seizure control in women with epilepsy on first-line antiepileptic drugs | No sufficient data |
| Ling, 2011[15] | Association of MDR1 gene tagSNPs rs3789243 and rs2235046 polymorphism with intractable epilepsy | Containing overlapping data |
| Hung, 2012[16] | Association of polymorphisms in EPHX1, UGT2B7 ABCB1, ABCC2, SCN1A and SCN2A genes with carbamazepine therapy optimization | Investigation of dose of AEDs |
| Dong, 2011[17] | Association between ABCB1 C3435T and drug-resistant epilepsy in Ningxia Hui epilepsy patients | Containing overlapping data |
| Hung, 2012[18] | Effects of polymorphisms in six candidate genes on phenytoin maintenance therapy in Han Chinese patients | Investigation of dose of AEDs |
| Lovric, 2012[19] | Association between lamotrigine concentrations and ABCB1 polymorphisms in patients with epilepsy | Investigation of concentrations AEDs |
| Ge, 2009[20] | Association between the C1236T polymorphism in multi-drug resistance gene 1 and response to antiepileptic drug treatment in epileptic patients | Containing overlapping data |

**References:**

1. Zimprich F, Sunder-Plassmann R, Stogmann E, Gleiss A, Dal-Bianco A, et al. (2004) Association of an ABCB1 gene haplotype with pharmacoresistance in temporal lobe epilepsy. Neurology 63: 1087-1089.

2. Leschziner G, Zabaneh D, Pirmohamed M, Owen A, Rogers J, et al. (2006) Exon sequencing and high resolution haplotype analysis of ABC transporter genes implicated in drug resistance. Pharmacogenet Genomics 16: 439-450.

3. Haerian BS, Lim KS, Tan HJ, Mohamed EH, Tan CT, et al. (2011) Association between ABCB1 polymorphism and response to sodium valproate treatment in Malaysian epilepsy patients. Epileptic Disord 13: 65-75.

4. Dericioglu N, Babaoglu MO, Yasar U, Bal IB, Bozkurt A, et al. (2008) Multidrug resistance in patients undergoing resective epilepsy surgery is not associated with C3435T polymorphism in the ABCB1 (MDR1) gene. Epilepsy Res 80: 42-46.

5. Mosyagin I, Runge U, Schroeder HW, Dazert E, Vogelgesang S, et al. (2008) Association of ABCB1 genetic variants 3435C>T and 2677G>T to ABCB1 mRNA and protein expression in brain tissue from refractory epilepsy patients. Epilepsia 49: 1555-1561.

6. Alpman A, Ozkinay F, Tekgul H, Gokben S, Pehlivan S, et al. (2010) Multidrug resistance 1 (MDR1) gene polymorphisms in childhood drug-resistant epilepsy. J Child Neurol 25: 1485-1490.

7. Dong L, Yu D, Mao M, Luo R, Cai X, et al. (2010) Correlation between C1236T polymorphism in ABCB1 gene and response of antiepileptic drug treatment in Chinese epileptic children. J Appl Clin Pediatr 25: 844-847.

8. Hung CC, Chen CC, Lin CJ, Liou HH (2008) Functional evaluation of polymorphisms in the human ABCB1 gene and the impact on clinical responses of antiepileptic drugs. Pharmacogenet Genomics 18: 390-402.

9. Ponnala S, Chaudhari JR, Jaleel MA, Bhiladvala D, Kaipa PR, et al. (2012) Role of MDR1 C3435T and GABRG2 C588T gene polymorphisms in seizure occurrence and MDR1 effect on anti-epileptic drug (phenytoin) absorption. Genet Test Mol Biomarkers 16: 550-557.

10. Guo G, Guo Y, Lin W, Meng H (2010) Association study of C3435T genetic polymorphism ofMDR1 gene with efficacy of carbamazepine. J Apoplexy and Nervous Diseases 27: 128-130.

11. Tate SK, Depondt C, Sisodiya SM, Cavalleri GL, Schorge S, et al. (2005) Genetic predictors of the maximum doses patients receive during clinical use of the anti-epileptic drugs carbamazepine and phenytoin. Proc Natl Acad Sci U S A 102: 5507-5512.

12. Wang G, Yang Z (2008) The relationship between the single nucleotide polymorphisms of multidrug resistance gene 1, brain derived neurotrophic factor gene and childhood drug resistance epilepsy. Chinese Pharmacological Bulletin 24: 933-937.

13. Puranik YG, Birnbaum AK, Marino SE, Ahmed G, Cloyd JC, et al. (2013) Association of carbamazepine major metabolism and transport pathway gene polymorphisms and pharmacokinetics in patients with epilepsy. Pharmacogenomics 14: 35-45.

14. Grover S, Gourie-Devi M, Bala K, Sharma S, Kukreti R (2012) Genetic association analysis of transporters identifies ABCC2 loci for seizure control in women with epilepsy on first-line antiepileptic drugs. Pharmacogenet Genomics 22: 447-465.

15. Ling L, Li L, Zhu Y, Mu Y, Zhou N, et al. (2011) Association of MDR1 gene tagSNPs rs3789243 and rs2235046 polymorphism with intractable epilepsy. Basic ＆ Clinical Medicine 31: 991-995.

16. Hung CC, Chang WL, Ho JL, Tai JJ, Hsieh TJ, et al. (2012) Association of polymorphisms in EPHX1, UGT2B7, ABCB1, ABCC2, SCN1A and SCN2A genes with carbamazepine therapy optimization. Pharmacogenomics 13: 159-169.

17. Dong T, Xu R, Zhang Q (2011) Association between ABCB1 C3435T and drug-resistant epilepsy in Ningxia Hui epilepsy patients. Journal of Ningxia Medical University 33: 108-110.

18. Hung CC, Huang HC, Gao YH, Chang WL, Ho JL, et al. (2012) Effects of polymorphisms in six candidate genes on phenytoin maintenance therapy in Han Chinese patients. Pharmacogenomics 13: 1339-1349.

19. Lovric M, Bozina N, Hajnsek S, Kuzman MR, Sporis D, et al. (2012) Association between lamotrigine concentrations and ABCB1 polymorphisms in patients with epilepsy. Ther Drug Monit 34: 518-525.

20. Ge L, Jin R, Wang J, Xu X, Li K (2009) Association between the C1236T polymorphism in multi-drug resistance gene 1 and response to antiepileptic drug treatment in epileptic patients. Journal of Shandong University (Health sciences) 47: 99-102.
