# Supplementary material for: The Roles of Variants in Human Multidrug Resistance (MDR1) Gene and Their Haplotypes on Antiepileptic Drugs Response: A Meta-Analysis of 57 Studies
Source: PLoS One. 2015 Mar 27;10(3):e0122043. doi: 10.1371/journal.pone.0122043 (PMC4376792; doi:10.1371/journal.pone.0122043)
Supplement: S2 Table — (DOC) [file pone.0122043.s003.doc]

**S2_Table**. Distribution of allelic frequencies (T allele in C1236T variant, T allele in G2677T variant, and T allele in C3435T variant) across different ethnic groups.

| **Variable** | **DNR** | **P** | **DR** | **P** | **Total patients** | **P** |
| --- | --- | --- | --- | --- | --- | --- |
| Frequency of T allelic in C3435T variant | | | | | | |
| Caucasians | 49.75%±8.24% | P_a_<0.01 | 54.89%±6.55% | P_a_<0.01 | 52.07%±5.88% | P_a_=<0.01 |
| Asians | 40.47%±8.09% | P_b_<0.01 | 43.83%±9.61% | P_b_=0.29 | 41.86%±6.44% | P_b_=0.02 |
| Indian | 61.40%±11.39% | P_c_<0.01 | 58.65%±10.43% | P_c_<0.01 | 60.28%±10.20% | P_c_=<0.01 |
| Frequency of T allelic in G2677T variant | | | | | | |
| Caucasians | 48.30%±11.45% | P_a_=0.87 | 47.73%%±6.53% | P_a_=0.24 | 47.49%±6.65% | P_a_=0.50 |
| Asians | 49.26±13.38% | P_b_<0.01 | 50.96%±5.21% | P_b_<0.01 | 49.83%±8.42% | P_b_<0.01 |
| Indian | 63.33%±2.64% | P_c_<0.01 | 61.50%±6.96% | P_c_<0.01 | 62.35%±4.91% | P_c_<0.01 |
| Frequency of T allelic in C1236T variant | | | | | | |
| Caucasians | 44.85%±7.88% | P_a_=0.01 | 52.50%±8.83% | P_a_=0.06 | 49.60%±7.22% | P_a_<0.01 |
| Asians | 63.46%±4.96% | P_b_<0.01 | 62.58%±3.40% | P_b_=0.10 | 62.90%±3.72% | P_b_=0.03 |
| Indian | 58.95%±4.85% | P_c_=0.12 | 60.47%±3.99% | P_c_=0.30 | 59.79%±4.23% | P_c_=0.17 |

DNR: Drug resistance; DR: Drug responsiveness; P_a_: P-value for Caucasians vs Asians; P_b_: P-value for Caucasians vs Indian; P_c_: P-value for Asians vs Indian
