# Supplementary material for: The Roles of Variants in Human Multidrug Resistance (MDR1) Gene and Their Haplotypes on Antiepileptic Drugs Response: A Meta-Analysis of 57 Studies
Source: PLoS One. 2015 Mar 27;10(3):e0122043. doi: 10.1371/journal.pone.0122043 (PMC4376792; doi:10.1371/journal.pone.0122043)
Supplement: S3 Table — (DOC) [file pone.0122043.s004.doc]

**S3_Table.** Summary odds ratios and heterogeneity of the G2677T polymorphism in ABCB1 gene on drug response in patients with epilepsy stratified by age, ethnicity, sample size and date of publication

|  | **No** | **T vs G** | | | | **TT vs GG** | | | | **TG vs GG** | | | | **TT+TG vs GG** | | | | **TT vs TG+GG** | | |
| --- | --- | --- | --- | --- | --- | --- | --- | --- | --- | --- | --- | --- | --- | --- | --- | --- | --- | --- | --- | --- |
|  |  | OR(95%CI) | P | P_h_ |  | OR(95%CI) | P | P_h_ |  | OR(95%CI) | P | P_h_ |  | OR(95%CI) | P | P_h_ |  | OR(95%CI) | P | P_h_ |
| **Total** | 25 | 0.95(0.80,1.12) | 0.52 | <0.01 | | 0.94(0.71,1.24) | 0.66 | <0.01 | | 0.87(0.68,1.11) | 0.27 | <0.01 | | 0.89(0.69,1.15) | 0.38 | <0.01 | | 0.98(0.82,1.18) | 0.83 | <0.01 |
| **All in HWE** | 18 | 1.04(0.94,1.16) | 0.41 | 0.15 | | 1.13(0.92,1.39) | 0.25 | 0.21 | | 1.00(0.84,1.19) | 0.97 | 0.30 | | 1.04(0.88,1.23) | 0.66 | 0.27 | | 1.09(0.93,1.28) | 0.29 | 0.18 |
| **Ethnicity** |  |  |  |  | |  |  |  | |  |  |  | |  |  |  | |  |  |  |
| Caucasians | 10 | 0.92(0.76,1.11) | 0.39 | 0.08 | | 0.83(0.57,1.20) | 0.32 | 0.13 | | 0.78(0.62,0.99) | 0.04 | 0.44 | | 0.79(0,61,1.04) | 0.09 | 0.21 | | 0.95(0.73,1.25) | 0.73 | 0.31 |
| Asians | 10 | 0.90(0.65,1.27) | 0.56 | <0.01 | | 0.81(0.52,1.28) | 0.37 | <0.01 | | 0.80(0.54,1.20) | 0.28 | <0.01 | | 0.85(0.52,1.38) | 0.51 | <0.01 | | 0.97(0.68,1.39) | 0.87 | <0.01 |
| Indian | 5 | 1.05(0.89,1.23) | 0.57 | 0.40 | | 1.26(0.85,1.87) | 0.25 | 0.27 | | 1.62(1.08,2.44) | 0.02 | 0.89 | | 1.23(0.84,1.79) | 0.29 | 0.27 | | 0.98(0.79,1.23) | 0.88 | 0.51 |
| **Age** |  |  |  |  | |  |  |  | |  |  |  | |  |  |  | |  |  |  |
| Children | 6 | 1.14(0.95,1.36) | 0.16 | 0.51 | | 1.31(0.92,1.87) | 0.13 | 0.50 | | 1.26(0.92,1.72) | 0.14 | 0.88 | | 1.27(0.95,1.70) | 0.10 | 0.82 | | 1.09(0.82,1.45) | 0.54 | 0.43 |
| Adults | 9 | 0.78(0.53,1.15) | 0.21 | <0.01 | | 0.62(0.35,1.11) | 0.11 | <0.01 | | 0.57(0.32,1.02) | 0.06 | <0.01 | | 0.60(0.33,1.08) | 0.09 | <0.01 | | 0.81(0.55,1.21) | 0.31 | <0.01 |
| **Sample size** |  |  |  |  | |  |  |  | |  |  |  | |  |  |  | |  |  |  |
| >200 | 18 | 0.90(0.75,1.09) | 0.28 | <0.01 | | 0.88(0.65,1.20) | 0.42 | <0.01 | | 0.88(0.67,1.16) | 0.37 | <0.01 | | 0.87(0.66,1.17) | 0.36 | <0.01 | | 0.92(0.75,1.12) | 0.41 | <0.01 |
| ≤200 | 7 | 1.14(0.83,1.55) | 0.42 | 0.08 | | 1.28(0.64,2.54) | 0.48 | 0.10 | | 0.82(0.46,1.47) | 0.51 | 0.15 | | 0.97(0.53,1.77) | 0.91 | 0.08 | | 1.37(0.91,2.07) | 0.13 | 0.31 |
| **Publication years** |  |  |  |  | |  |  |  | |  |  |  | |  |  |  | |  |  |  |
| >2010 | 11 | 0.82(0.59,1.14) | 0.25 | <0.01 | | 0.75(0.47,1.20) | 0.23 | <0.01 | | 0.69(0.42,1.13) | 0.14 | <0.01 | | 0.72(0.44,1.18) | 0.20 | <0.01 | | 0.86(0.64,1.16) | 0.34 | <0.01 |
| ≤2010 | 14 | 1.06(0.93,1.20) | 0.37 | 0.10 | | 1.13(0.85,1.50) | 0.40 | 0.05 | | 1.03(0.84,1.25) | 0.79 | 0.30 | | 1.06(0.87,1.29) | 0.59 | 0.18 | | 1.09(0.88,1.35) | 0.43 | 0.07 |

CI: confidence interval; HWE: Hardy-Weinberg equilibrium; No: Number of studies; OR: odds ratio; P_h_: P-value for heterogeneity tests.
