# Supplementary material for: The Roles of Variants in Human Multidrug Resistance (MDR1) Gene and Their Haplotypes on Antiepileptic Drugs Response: A Meta-Analysis of 57 Studies
Source: PLoS One. 2015 Mar 27;10(3):e0122043. doi: 10.1371/journal.pone.0122043 (PMC4376792; doi:10.1371/journal.pone.0122043)
Supplement: S4 Table — (DOC) [file pone.0122043.s005.doc]

**S4_Table.** Summary odds ratios and heterogeneity of the C1236T polymorphism in ABCB1 gene on drug response in patients with epilepsy stratified by age, ethnicity, sample size and date of publication

|  | **No** | **T vs G** | | | | **TT vs CC** | | | | **TC vs CC** | | | | **TT+TC vs CC** | | | | **TT vs TC+CC** | | |
| --- | --- | --- | --- | --- | --- | --- | --- | --- | --- | --- | --- | --- | --- | --- | --- | --- | --- | --- | --- | --- |
|  |  | OR(95%CI) | P | P_h_ |  | OR(95%CI) | P | P_h_ |  | OR(95%CI) | P | P_h_ |  | OR(95%CI) | P | P_h_ |  | OR(95%CI) | P | P_h_ |
| **Total** | 20 | 0.97(0.90,1.06) | 0.56 | 0.51 | | 0.96(0.81,1.14) | 0.67 | 0.69 | | 1.01(0.86,1.20) | 0.88 | 0.81 | | 0.99(0.84,1.16) | 0.88 | 0.75 | | 0.97(0.86,1.09) | 0.63 | 0.65 |
| **All in HWE** | 16 | 0.99(0.90,1.09) | 0.85 | 0.45 | | 0.98(0.81,1.19) | 0.87 | 0.68 | | 1.04(0.86,1.25) | 0.68 | 0.83 | | 1.01(0.85,1.20) | 0.88 | 0.77 | | 0.97(0.86,1.11) | 0.71 | 0.51 |
| **Ethnicity** |  |  |  |  | |  |  |  | |  |  |  | |  |  |  | |  |  |  |
| Caucasians | 5 | 0.82(0.66,1.00) | 0.06 | 0.24 | | 0.70(0.44,1.11) | 0.13 | 0.24 | | 0.94(0.62,1.42) | 0.76 | 0.18 | | 0.84(0.57,1.25) | 0.40 | 0.13 | | 0.73(0.51,1.04) | 0.08 | 0.53 |
| Asians | 10 | 1.04(0.93,1.16) | 0.47 | 0.54 | | 1.05(0.83,1.32) | 0.69 | 0.70 | | 0.99(0.79,1.24) | 0.92 | 0.81 | | 1.01(0.82,1.25) | 0.90 | 0.80 | | 1.07(0.92,1.25) | 0.36 | 0.64 |
| Indian | 5 | 0.94(0.81,1.11) | 0.48 | 0.82 | | 0.95(0.69,1.31) | 0.76 | 0.81 | | 1.11(0.81,1.52) | 0.52 | 0.71 | | 1.03(0.77,1.38) | 0.86 | 0.73 | | 0.88(0.70,1.10) | 0.26 | 0.85 |
| **Age** |  |  |  |  | |  |  |  | |  |  |  | |  |  |  | |  |  |  |
| Children | 5 | 0.91(0.76,1.09) | 0.31 | 0.54 | | 0.80(0.56,1.14) | 0.21 | 0.64 | | 0.89(0.63,1.25) | 0.50 | 0.37 | | 0.86(0.63,1.19) | 0.37 | 0.44 | | 0.91(0.70,1.18) | 0.49 | 0.72 |
| Adults | 6 | 0.95(0.81,1.11) | 0.54 | 0.10 | | 0.93(0.65,1.33) | 0.71 | 0.14 | | 0.94(0.66,1.32) | 0.72 | 0.45 | | 0.92(0.66,1.27) | 0.61 | 0.21 | | 0.99(0.78,1.26) | 0.94 | 0.25 |
| **Sample size** |  |  |  |  | |  |  |  | |  |  |  | |  |  |  | |  |  |  |
| >200 | 15 | 0.99(0.91,1.08) | 0.84 | 0.51 | | 0.98(0.82,1.18) | 0.86 | 0.66 | | 1.04(0.87,1.24) | 0.68 | 0.80 | | 1.01(0.86,1.20) | 0.86 | 0.74 | | 0.97(0.86,1.10) | 0.68 | 0.56 |
| ≤200 | 5 | 0.85(0.66,1.10) | 0.21 | 0.44 | | 0.74(0.40,1.37) | 0.34 | 0.47 | | 0.78(0.44,1.38) | 0.40 | 0.48 | | 0.75(0.44,1.27) | 0.29 | 0.49 | | 0.92(0.58,1.46) | 0.74 | 0.46 |
| **Publication years** |  |  |  |  | |  |  |  | |  |  |  | |  |  |  | |  |  |  |
| >2010 | 7 | 0.91(0.79,1.05) | 0.21 | 0.61 | | 0.85(0.64,1.14) | 0.27 | 0.69 | | 0.93(0.71,1.24) | 0.64 | 0.65 | | 0.89(0.68,1.15) | 0.37 | 0.63 | | 0.89(0.73,1.09) | 0.28 | 0.78 |
| ≤2010 | 13 | 1.01(0.91,1.12) | 0.96 | 0.41 | | 1.03(0.83,1.28) | 0.77 | 0.57 | | 1.06(0.86,1.31) | 0.59 | 0.70 | | 1.05(0.86,1.28) | 0.63 | 0.68 | | 1.02(0.87,1.18) | 0.83 | 0.45 |

CI: confidence interval; HWE: Hardy-Weinberg equilibrium; No: Number of studies; OR: odds ratio; P_h_: P-value for heterogeneity tests.
