# Supplementary material for: The Roles of Variants in Human Multidrug Resistance (MDR1) Gene and Their Haplotypes on Antiepileptic Drugs Response: A Meta-Analysis of 57 Studies
Source: PLoS One. 2015 Mar 27;10(3):e0122043. doi: 10.1371/journal.pone.0122043 (PMC4376792; doi:10.1371/journal.pone.0122043)
Supplement: S5 Table — (DOC) [file pone.0122043.s006.doc]

**S5_Table.** Distribution of haplotypic frequencies of the ACBC1 polymorphisms at C1236T, G2677T and C3435T.

| **Haplotype** | Seo, 2006  Asians | | Kim, 2006  Asians | | Lakhan, 2009  Indian | | Vahab, 2009  Indian | | Grover, 2009  Indian | | Hung, 2005  Asians | | Dong,2011  Asians | | Haerian,2011  Asians | | Haerian,2011  Indian | | Haerian,2011  Asians | |
| --- | --- | --- | --- | --- | --- | --- | --- | --- | --- | --- | --- | --- | --- | --- | --- | --- | --- | --- | --- | --- |
| 1236-2677-3435 | DNR | DR | DNR | DR | DNR | DR | DNR | DR | DNR | DR | DNR | DR | DNR | DR | DNR | DR | DNR | DR | DNR | DR |
| Total | 252 | 168 | 194 | 198 | 188 | 462 | 226 | 258 | 190 | 266 | 216 | 446 | 314 | 386 | 262 | 292 | 134 | 186 | 250 | 246 |
| C-G-C | 38 | 33 | 39 | 34 | 43 | 89 | 19 | 35 | 56 | 59 | 76 | 18 | 64 | 81 | 45 | 43 | 27 | 55 | 49 | 60 |
| T-T-T | 106 | 51 | 53 | 49 | 84 | 194 | 58 | 65 | 98 | 140 | 66 | 31 | 92 | 129 | 56 | 51 | 38 | 38 | 53 | 61 |
| C-G-T | 10 | 5 | - | - | 8 | 22 | 18 | 26 | 6 | 22 | 3 | 127 | 10 | 4 | 13 | 32 | 8 | 15 | 26 | 16 |
| T-G-C | 40 | 34 | 41 | 39 | 4 | 9 | 35 | 28 | - | - | 53 | 4 | 71 | 89 | 40 | 41 | 27 | 29 | 31 | 34 |
| C-T-T | 3 | 4 | 2 | 3 | 14 | 25 | 61 | 59 | 8 | 14 | 2 | 7 | 3 | 0 | 22 | 17 | 5 | 9 | 10 | 18 |
| T-G-T | 7 | 2 | 2 | 9 | 12 | 41 | 22 | 18 | - | - | 5 | 101 | 5 | 6 | 32 | 31 | 5 | 6 | 15 | 14 |
| T-T-C | 18 | 21 | 21 | 19 | 11 | 36 | 5 | 16 | 11 | 20 | 11 | 152 | 30 | 30 | 23 | 51 | 19 | 12 | 54 | 17 |
| C-T-C | 30 | 18 | 36 | 45 | 12 | 46 | 8 | 11 | - | - | 0 | 6 | 39 | 47 | 31 | 26 | 7 | 20 | 12 | 26 |
| Others | - | - | - | - | - | - | - | - | 11 | 11 | - | - | - | - | - | - | - | - | - | - |

DNR: Drug resistance; DR: Drug responsiveness.
