# Supplementary material for: The Roles of Variants in Human Multidrug Resistance (MDR1) Gene and Their Haplotypes on Antiepileptic Drugs Response: A Meta-Analysis of 57 Studies
Source: PLoS One. 2015 Mar 27;10(3):e0122043. doi: 10.1371/journal.pone.0122043 (PMC4376792; doi:10.1371/journal.pone.0122043)
Supplement: S6 Table — (DOC) [file pone.0122043.s007.doc]

**S6_Table**. Summary odds ratios and heterogeneity of haplotypic comparisons in ABCB1 gene on drug response in patients with epilepsy stratified by ethnicity

|  | No | TTT vs CGC | | |  | TTT vs non-TTT | | |  | Non-CGC vs CGC | | |
| --- | --- | --- | --- | --- | --- | --- | --- | --- | --- | --- | --- | --- |
|  |  | OR(95%CI) | P | P_h_ |  | OR(95%CI) | P | P_h_ |  | OR(95%CI) | P | P_h_ |
| Total |  | 1.04(0.82,1.32) | 0.72 | 0.04 |  | 1.31(0.94,1.81) | 0.11 | <0.01 |  | 0.83(0.51,1.35) | 0.46 | <0.01 |
| Ethnicity |  |  |  |  |  |  |  |  |  |  |  |  |
| Asians |  | 0.99(0.74,1.33) | 0.96 | 0.13 |  | 1.45(0.83,2.52) | 0.19 | <0.01 |  | 0.69(0.32,1.48) | 0.34 | <0.01 |
| Indian |  | 1.15(0.73,1.83) | 0.53 | 0.03 |  | 1.10(0.90,1.34) | 0.35 | 0.52 |  | 1.08(0.68,1.71) | 0.74 | 0.01 |

CI: confidence interval; No: Number of studies; OR: odds ratio; P_h_: P-value for heterogeneity tests.
