# Supplementary figures and images for: The Roles of Variants in Human Multidrug Resistance (MDR1) Gene and Their Haplotypes on Antiepileptic Drugs Response: A Meta-Analysis of 57 Studies
Source: PLoS One. 2015 Mar 27;10(3):e0122043. doi: 10.1371/journal.pone.0122043 (PMC4376792; doi:10.1371/journal.pone.0122043)

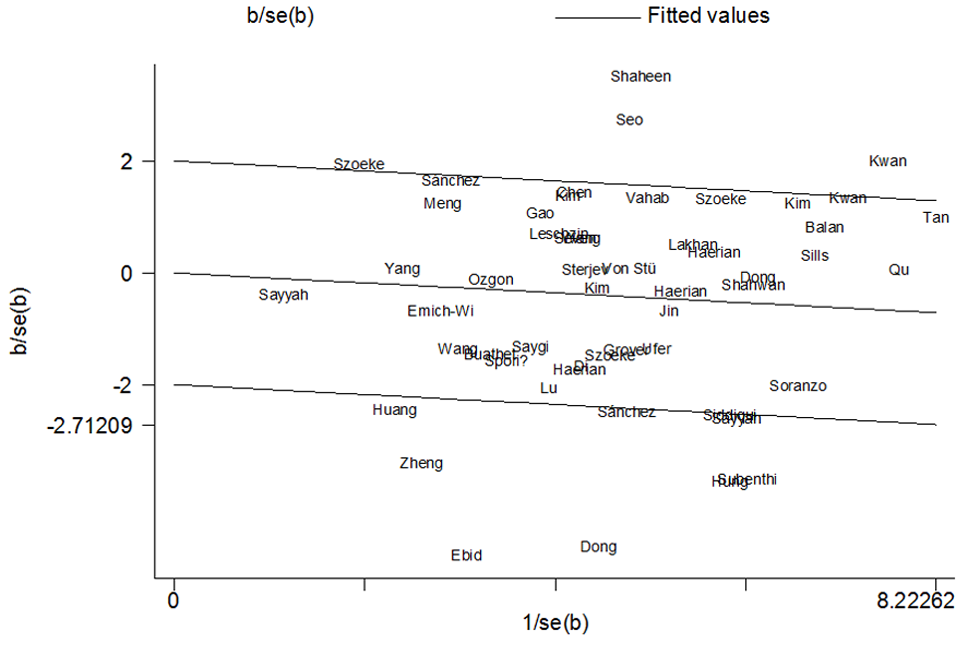

Supplement: S1 Fig — The 95% confidence interval is between the two outer parallel lines at two units above and below the regression line. (TIF) [file pone.0122043.s008.tif]

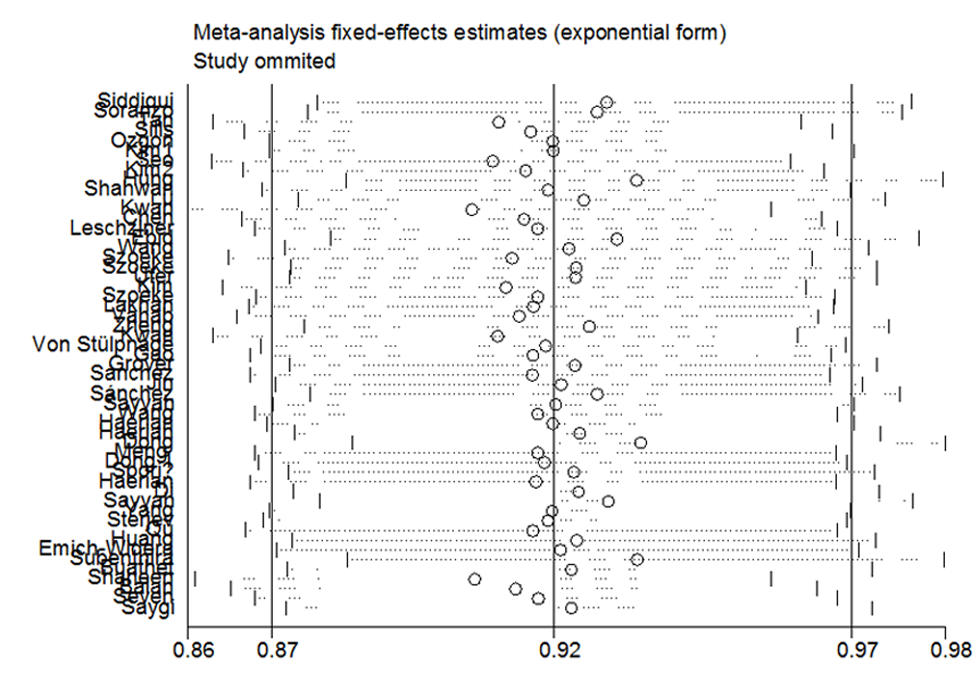

Supplement: S2 Fig — Results were computed by omitting each study (left column) in turn, Bars: 95% confidence interval. (TIF) [file pone.0122043.s009.tif]

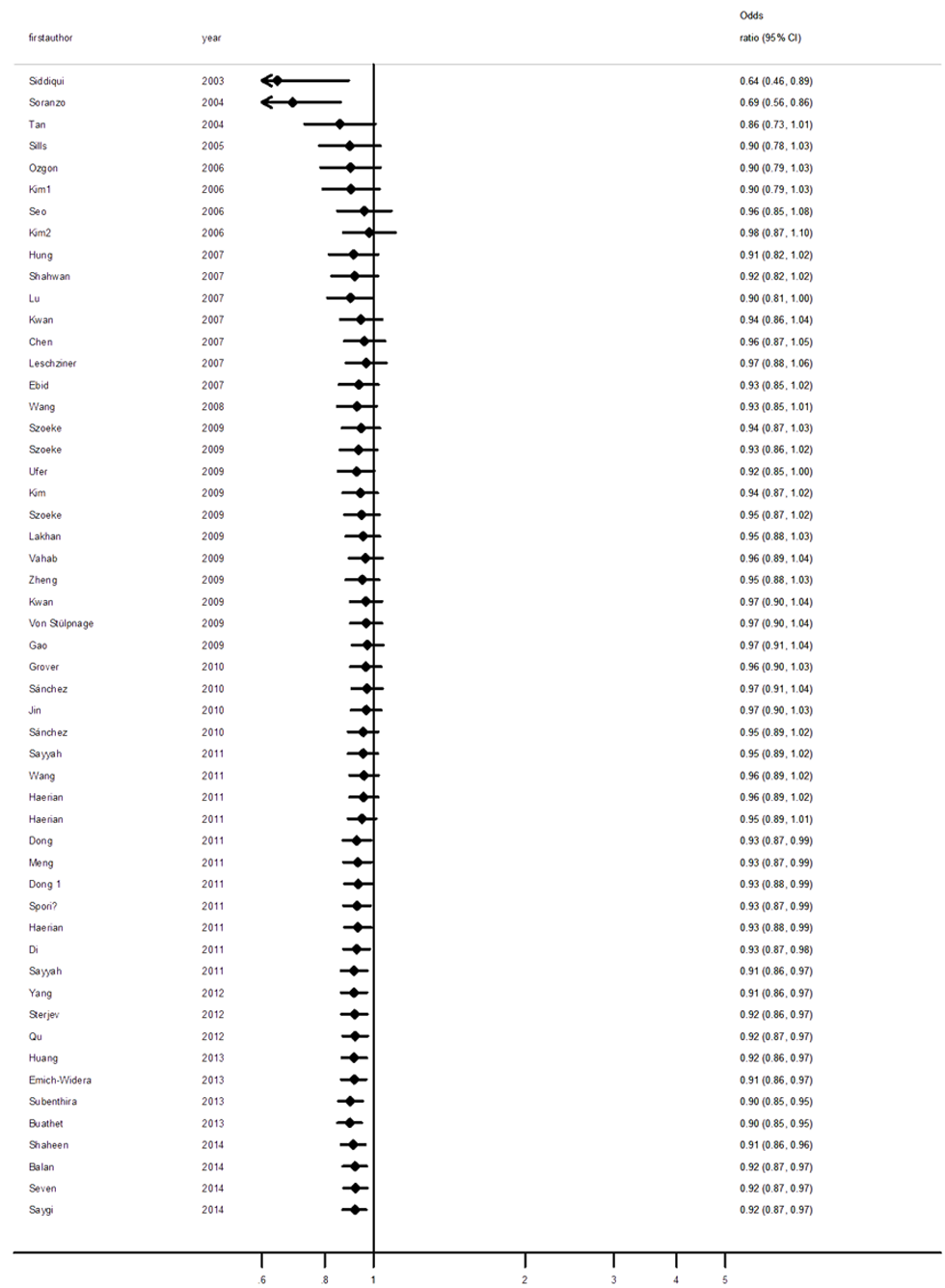

Supplement: S3 Fig — The circles and horizontal lines show the accumulation of estimates as results from each study at the end of each year were added. (TIF) [file pone.0122043.s010.tif]
